# Invariant Natural Killer T Cells in Immune Regulation of Blood Cancers: Harnessing Their Potential in Immunotherapies

**DOI:** 10.3389/fimmu.2017.01355

**Published:** 2017-10-23

**Authors:** Pui Yeng Lam, Michael D. Nissen, Stephen R. Mattarollo

**Affiliations:** ^1^The University of Queensland Diamantina Institute, The University of Queensland, Translational Research Institute, Brisbane, QLD, Australia

**Keywords:** invariant natural killer T, natural killer T cells, blood cancer, immunosurveillance, immunotherapy, tumor immune evasion

## Abstract

Invariant natural killer T (iNKT) cells are a unique innate T lymphocyte population that possess cytolytic properties and profound immunoregulatory activities. iNKT cells play an important role in the immune surveillance of blood cancers. They predominantly recognize glycolipid antigens presented on CD1d, but their activation and cytolytic activities are not confined to CD1d expressing cells. iNKT cell stimulation and subsequent production of immunomodulatory cytokines serve to enhance the overall antitumor immune response. Crucially, the activation of iNKT cells in cancer often precedes the activation and priming of other immune effector cells, such as NK cells and T cells, thereby influencing the generation and outcome of the antitumor immune response. Blood cancers can evade or dampen iNKT cell responses by downregulating expression of recognition receptors or by actively suppressing or diverting iNKT cell functions. This review will discuss literature on iNKT cell activity and associated dysregulation in blood cancers as well as highlight some of the strategies designed to harness and enhance iNKT cell functions against blood cancers.

## Introduction

Blood cancers are a heterogeneous group of malignancies broadly encompassing leukemia, myeloma, and lymphoma. As these cancers develop largely in lymphoid tissues, immune surveillance mechanisms are engaged, but inevitably fail due to changes in the microenvironment which are permissive to tumor growth but impede the development of antitumor immunity. Invariant natural killer T (iNKT) cells, an innate-like lymphocyte population defined by their semi-invariant T cell receptor (TCR)—Vα14Jα18 in mice and Vα24Jα18 in humans, have important roles in helping to regulate antitumor responses to cancer (1). These cells share similar properties to that of NK and T cells. The discovery of a potent prototypical NKT cell-activating glycolipid ligand known as α-galactosylceremide (αGalCer) (2, 3) prompted extensive attempts to manipulate this population to enhance antitumor immunity, both in solid and blood cancers. This review focuses on the activities of iNKT cells in blood malignancies and discusses the potential avenues for therapeutic targeting of iNKT cells in humans based on preclinical evidence (Table 1).

**Table 1:** Evidence for the involvement and effective targeting of iNKT cells for blood cancer control in mice and humans.

Blood cancer type	Mouse	Human
Lymphoma	CD1d^+^ tumors can be recognized by NKT cells *in vitro* (4) Altered glycosphingolipids secreted by T lymphoma cell line shield iNKT cell recognition (5) αGalCer-pulsed tumor cells ± checkpoint agonist provide protection (6, 7) Pulsing of DCs with αGalCer and tumor antigen provides protection (ATOO) (8) Adoptive transfer of *ex vivo* activated iNKT cells provides protection (ALC) (9) NKT cells transduced with CD62L CAR persist *in vivo* and prevents tumor growth (10) DC-targeted nanoparticle provides prophylactic and therapeutic protection (11)	Frequency of iNKT cells varies between loci of disease, disease stage, and subtypes (12, 13) CIK cells activated and expanded *ex vivo* show partial clinical efficacy against advanced lymphoma [reviewed (14, 15)]
Acute/chronic myeloid leukemia	αGalCer-pulsed tumor cells provide protection (7)	Low expression of CD1d correlate with poorer prognosis (16) Functional defects in NKT cells and CD1d downregulation induced by oncogene expression (17, 18) Tyrosine kinase inhibitor can restore iNKT cell functions (17) Activated iNKT cells is cytotoxic against CD1d^+^ tumor cells *in vitro* (19, 20)
Acute lymphocytic leukemia	αGalCer-pulsed tumor cells provide protection prophylactically. Therapeutic vaccine combined with chemotherapy is protective (C1498) (21) NKT-like cells transduced with CD19-directed CAR is protective and promotes long term survival (22)	Low expression of CD1d may contribute to progression (16), yet CD1d^+^ leukemia can also associate with poor prognosis (23) CIK cells transduced with CD19-directed CAR kill tumor cells *in vitro* (22)
Chronic lymphocytic leukemia	CD1d-deficiency shortens survival (TCL1) (24) NKT cells delay disease onset but become functionally impaired	Reduced frequency, function and expression of CD1d on tumors is associated with progression of disease (13, 24–28) Higher CD1d expression can also be associated with poor prognosis (27, 29) Higher presentation of tumor-associated lipids on CD1d can lead to impairment of CD3ζ signaling and poorer prognosis (29) Cultured iNKT-like/CIK cells are cytotoxic against tumor *in vitro* (30–33)
Multiple myeloma	αGalCer-pulsed DCs improves survival outcome of mice (5T33MM) (34) αGalCer-pulsed tumor cells provides protection (Vk*myc, MOPC315.BM) (7, 35)	Reduced frequency and function of iNKT cells correlates with disease progression (36) Inflammation associated lipids skew Th2 responses in iNKT cells (36, 37) Cultured expanded NKT cells are cytotoxic against CD1d^+^ myeloma cells *in vitro* (20, 36) αGalCer-pulsed DCs ± lenalidomide induce NKT cell expansion (38, 39)

## Immunoregulatory and Direct Cytotoxic Activities of iNKT Cells in Blood Cancers

Invariant natural killer T cells recognize glycolipid antigens presented on the MHC Class I-like molecule CD1d, which are expressed on many cell types, but most highly expressed on antigen-presenting cells (APCs) (40, 41). Both human and murine iNKT cells were found to recognize glycolipid antigens derived from components of bacteria (42, 43), as well as the synthetic molecule, αGalCer (44). However, iNKT cells have also been shown to recognize and respond to a variety of endogenous lipids including lysosomal glycosphingolipids such as isoglobotrihexosylceramide (iGb3) (45–48). iNKT cells were shown to directly recognize and kill various human tumor cell lines *in vitro* and murine tumors *in vitro* and *in vivo* through the recognition of endogenous lipids expressed on CD1d (36, 49, 50). The identities of these tumor-associated lipid antigens are mostly unknown. However, the tumor-associated ganglioside GD3 can be presented on CD1d for the activation of iNKT cells *in vivo* (45).

**Figure 1:**
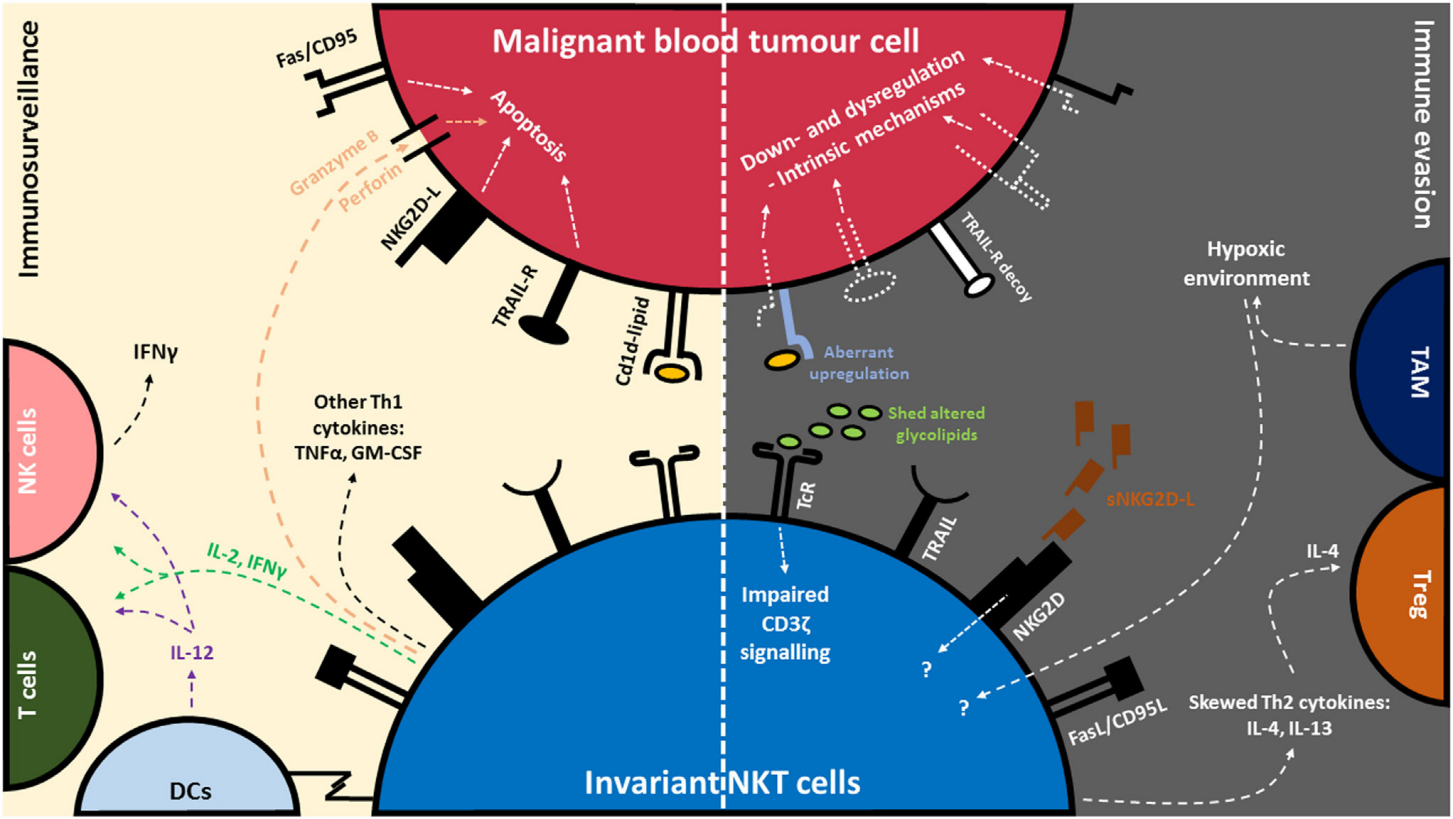
Invariant natural killer T (iNKT) cell-mediated immune surveillance of blood cancer and counteractive evasion strategies utilized by blood cancer cells. (Left) iNKT cells recognize glycolipid antigens presented on CD1d, commonly expressed by blood tumor cells. Recognition of glycolipid:CD1d complex *via* the invariant T cell receptor (TcR) leads to a cascade of events: the production of immunomodulatory cytokines such as interleukin-2 (IL-2), interferon-γ (IFNγ), tumor necrosis factor-α (TNFα), and granulocyte-macrophage colony-stimulating factor (GM-CSF), release of cytolytic mediators such as perforin/granzyme, activation of antigen-presenting cells (APCs) such as dendritic cells (DCs) and IL-12 production, as well as the rapid transactivation of NK cells and T cells. iNKT cells can also recognize tumor and degranulate in a CD1d-independent manner *via* Natural Killer Group 2D (NKG2D) receptors. (Right) In turn, tumor cells can evade recognition and killing by downregulating CD1d, NKG2D-L, TNF-related apoptosis-inducing ligand (TRAIL-L) and FAS/CD95. In addition, certain blood tumors can disrupt death signaling pathways to avoid killing. Some blood tumors express aberrant levels of glycolipids or shed soluble glycolipids and NKG2D-L which in turn dysregulate normal signaling pathway in iNKT cells. Blood tumors cells can also skew the production of Th2 cytokines (IL-4 and IL-13) in iNKT cells. IL-4 is associated with the activation of regulatory T cells (Treg) which are involved in dampening of antitumor responses. Dysfunction of iNKT cells have also been associated with tumor-associated macrophages (TAMs) and their ability to induce hypoxia in the tumor microenvironment.

Early preclinical studies demonstrated that engagement of lipid antigen-CD1d complexes *via* the iNKT TCR results in the production of a diverse range of Th1/Th2 cytokines and chemokines (51–53), which can subsequently modulate both innate and adaptive immune cells. Notably, activation of iNKT cells leads to the downstream activation of NK cells and enhanced IFNγ production (54, 55), dendritic cell (DC) maturation and IL-12 production, and the induction of CD4 and CD8 T cell responses (56–59). Consequently, this cascade of events constitutes the indirect antitumor immunity imparted by activated iNKT cells (transactivation). Indeed, mice lacking iNKT cells (CD1d^−/−^ and Jα18^−/−^ mice) are more susceptible to tumor development in several spontaneous, oncogenic and carcinogenic models (60–63). In recent years, several studies have established the direct and spontaneous role of iNKT cells in the initiation of innate immune responses against blood cancers such as B/T cell lymphomas, chronic lymphocytic leukemia (CLL) and multiple myeloma (MM) (25, 36, 64–66). These studies show that iNKT cells have the potential to control or delay the progression of premalignant or early stage disease in a CD1d-dependent manner, as seen using murine models and iNKT cells derived from patients (4, 19, 49, 67–69). In addition, innate immune control of blood cancers was found to correlate to the functional ability of iNKT cells to produce inflammatory cytokines IFNγ, and TNFα and as well as the induction of IL-12 production in APCs (64, 70, 71) (Figure 1).

In addition to their immunostimulatory effects, activated iNKT cells possess direct cytotoxic activity against blood cancers through the production of cytolytic molecules such as granzyme B and perforin, and through the interaction of death-inducing receptors such as Fas and TRAIL (19, 49, 72–75). More than half of all iNKT cells also express the NKG2D activating receptor enabling direct cytotoxicity against tumors expressing NKG2D ligands (76, 77). More broadly, NKG2D expression on immune effector cells is important for protection against hematological malignancy (78) (Figure 1). This was supported by two recent studies performed in NKG2D-deficient mice, which developed spontaneous lymphomas significantly faster than NKG2D-competent mice (79, 80). Similarly, the success of various inhibitors administered in mice that prevent the shedding of NKG2D ligands (NKG2D-L) or induce NKG2D-L expression on leukemic cells, and thereby enhancing cytotoxic killing, further demonstrates the significant role of NKG2D expression in immune surveillance of blood cancers (81, 82). In contrast, the functional role of NKG2D on human iNKT cells against tumors is less well defined. It has, however been demonstrated that human CD3^+^CD56^+^ NKT-like cells derived from the blood of healthy individuals are sensitive towards NKG2D-L-expressing cell lines including monocytic lymphoma (U937) and Burkitt’s lymphoma cell lines (Raji) (77, 83). More studies are required to understand the extent to which NKG2D expression on human iNKT cells is effective against blood cancers.

Invariant natural killer T cells have also been identified in the control of host response against allogenic donor cell rejection in leukemic patients receiving allogeneic HSCT. The suppression of graft-versus-host-disease (GvHD), while maintaining graft-versus-tumor effect has been shown to be highly dependent on the engraftment of donor iNKT cells, as failure to reconstitute iNKT cells after transplantation strongly correlated with disease relapse (84–87). Studies into the mechanisms of GvHD suppression show that iNKT cells modulate the overall immune response through production of Th2 cytokines such as IL-4, which in turn dampen inflammatory donor T cells, and promote Treg proliferation against both acute and chronic GvHD (88–91). These studies therefore highlight an importance function of the Th2 arm of activated iNKT cells in the facilitation of engraftment of allogenic donor cells against recurrence of leukemia.

## iNKT Cell Dysfunction and Evasion of iNKT Cell Recognition in Blood Cancers

### Tumor Cell Evasion of iNKT Cell Recognition and Killing

Blood tumor cells possess intricate methods of evading detection and elimination by the immune system (92–94). The downregulation of CD1d on malignant cells is one of the major contributing factors to the evasion of iNKT cell immunosurveillance in blood cancers (34, 95). In fact, lower expression levels of CD1d on a variety of blood cancers is associated with progressive and advanced stages of disease in both murine models and in humans (16, 25, 26, 64, 96). Various mechanisms have been associated with downregulation of CD1d expression in blood cancers. For example, surface CD1d downregulation in Epstein–Barr virus-transformed B cells is thought to be attributed to posttranscriptional mechanisms commonly employed by herpes viruses (97, 98). Downregulation of CD1d expression on CLL B cells is believed to be associated with the elevated levels of a transcriptional protein called lymphoid enhancer-binding factor-1 (26).

Aside from regulation of CD1d expression, blood cancers may also be able to evade recognition by NKG2D on iNKT cells. This assumption is derived from previous observations in solid tumors. In one particular study, serum samples taken from patients with ovarian and prostate cancer had elevated levels of tumor-derived soluble NKG2D ligands, namely MHC class I chain-related (MIC) proteins. When cocultured with freshly isolated iNKT-like CD3^+^CD56^+^ cells *in vitro*, the cytotoxic activity of these cells was compromised and NKG2D expression was downregulated (83). In a more recent study, Lu et al. (99) demonstrated that antibody blockade of soluble MIC in a model of adenocarcinoma could potentiate IFNγ production upon stimulation (100) As elevated levels of soluble NKG2D ligands in the plasma of patients with MM, acute lymphoblastic leukemia (ALL), chronic myeloid leukemia (CML), Hodgkin’s lymphoma (HL), and non-HL have been observed (101–105), it is predicted that NKG2D-expressing iNKT cells will be dysregulated in these tumor microenvironments. With evidence showing the capacity for iNKT cells to utilize TRAIL to kill leukemic cells *in vitro* (19), it is anticipated that blood tumors would be able to evade recognition by iNKT cells by altering TRAIL receptor expression. Indeed, myeloma and B cell lymphomas have been reported to resist TRAIL-induced killing (106), by downregulating TRAIL receptors—death receptor 4 (DR4) and DR5 (107, 108), or by dysregulating receptor signaling to evade killing (109, 110). Likewise, AML tumors have been observed to utilize decoy TRAIL receptors to resist apoptosis (111, 112).

### Immunosuppressive Effects of Tumors on iNKT Cells

Blood cancer disease progression in humans is associated with a profound decrease in the frequency and function of circulating iNKT cells (12, 113–119). Although iNKT cell numbers have been shown to vary between subtypes and grade of B cell neoplasms in humans (13), this parameter has been used as an independent factor for predicting disease stage and progression in blood cancer patients (25, 36, 118). It is currently unclear how disease progression causes these defects in iNKT cells. Several studies have suggested that iNKT cell dysfunction caused by tumors are indirect, as iNKT cell function and expansion can be rescued upon administration of αGalCer-based treatments (36, 67, 120, 121), or lenalidomide treatment (122, 123). In studies in CML patients, aberrant tyrosine kinase expression and dysfunctional Rho-associated protein kinase (ROCK) expression have been suggested to exert suppressive effects on iNKT cells by regulating the transcription factor PLZF, expression of CD95L and perforin (17) as well as altering CD1d expression on myeloid DCs (mDCs) (18). Indeed, in CML patients who had undergone treatment using a tyrosine kinase inhibitor, iNKT cell functions could be restored (17). Likewise, *in vitro* treatment of CML mDCs with ROCK inhibitors was found to partially restore CD1d expression (18). iNKT cell dysfunction has also been associated with tumor-associated lipid antigen production, such as altered glycosphingolipids secreted by a murine T cell lymphoma cell line. The shedding of these lipid antigens were suggested to shield from iNKT cell recognition, as inhibition of the release of these lipid antigens could rescue iNKT cell functions (5). Interestingly, in certain patients with leukemia, higher CD1d levels have been detected on malignant cells that correlated with poorer prognosis and lower iNKT cell numbers (23, 27, 29). In this instance, higher presentation of tumor-associated lipids on CD1d by leukemic cells was suggested to cause iNKT cell hyporesponsiveness attributed to an impairment of CD3ζ signaling (29). In MM patients, inflammation-associated lysophospholipids and other glycolipids found to be elevated in the plasma were shown to induce iNKT cells to produce the Th2 cytokine IL-13 (36, 37), an anti-inflammatory cytokine associated with downregulation of tumor immunosurveillance (124). iNKT cell dysfunction has also been linked to hypoxia and tumor-associated macrophages (125), as well as interruptions in metabolic signaling caused by acidity of the tumor microenvironment (126) (Figure 1). These conditions have been implied to promote lymphoma tumor progression (127, 128). Better understanding of these immunosuppressive strategies of blood cancers will help with designing strategies that better harness the antitumor effects of iNKT cells.

## Strategies to Modulate iNKT Cell Activity in Blood Cancers

### Early Use of iNKT Cell Adjuvants

Over the past couple of decades, strategies to exploit iNKT cells have been explored to treat various types of cancer, including blood cancers. Early studies in preclinical models showed that direct injection of αGalCer or its derivatives could induce potent iNKT cell activation and subsequent innate and adaptive immune suppression of tumors, but was also associated with significant liver toxicity (63, 71, 129, 130). Unfortunately however, this antitumor effect was not recapitulated when tested against human cancers. A phase I clinical trial using αGalCer instead found limited value as a direct immunotherapeutic agent against advanced solid cancers, despite a relatively safe toxicity profile tested in dose-escalating studies (131, 132). Patients with a higher frequency of circulating iNKTs did however respond better to treatment and produce enhanced immunological responses (133). Yet, the induction of immunological activity in these patients did not result in any partial or complete responses, and only disease stabilization in some patients could be achieved (131, 132).

### DC Vaccines

Subsequently, it was revealed that free-form αGalCer causes profound and enduring hyporesponsiveness in iNKT cells (134, 135). To overcome this treatment-induced anergy, various other delivery strategies have been designed, including the *ex vivo* stimulation and loading of autologous DCs with αGalCer. Initial studies in solid tumor preclinical models showed that administration of αGalCer-pulsed DCs could enhance the frequency of iNKT cells and circulating IFNγ-producing cells, as well as Th1 antitumor responses when compared to free-form αGalCer (38, 136, 137). In addition, αGalCer-pulsed DCs can also efficiently promote the infiltration of lymphocytes including iNKT cells into tumors, enhance circulating levels of IFNγ (138, 139), and promote iNKT cell-induced immune memory upon secondary administration (140). These properties are believed to contribute in part to the long-term survival of tumor-bearing mice receiving DC therapy. For example, αGalCer-pulsed DCs has been shown to improve overall survival of mice with MM (5T33MM model) (34). When tested in patients with advanced MM, administration of αGalCer-pulsed DCs was found to sufficiently induce iNKT cell expansion and persistence in the blood (38). However, this study did not observe any overall clinical improvement in these patients. In a Phase I/II study in six patients with asymptomatic myeloma, the combination therapy of αGalCer-pulsed monocyte-derived DCs with low-dose lenalidomide, resulted in improved modulation of both iNKT and NK cell responses, including the increased surface expression of NKG2D on NK cells. The addition of lenalidomide was intended to augment the effects of DC vaccination (39), as lenalidomide have been previously suggested to skew iNKT cell and cytokine induced killer (CIK) cell responses toward a protective Th1 profile in MM patients (123, 141, 142). Similarly, coloading of DCs with αGalCer and irradiated tumor cells has also been shown to be highly protective against B cell lymphoma in mice (4TOO model) (8). In this instance, the pulsing of DCs with tumor cells served to provide a source of undefined tumor antigens to initiate tumor-specific immune responses enhanced by the adjuvanting effects of αGalCer.

### Tumor Cell-Based Vaccines

We and others have previously attempted to use autologous tumor cells as vaccine vehicles for αGalCer delivery in mice. Single administration of an αGalCer-loaded tumor cell vaccine could induce potent antitumor immunity and prolong overall survival in mice with various blood cancers, including B lymphoma (Eμ-myc), acute myeloid leukemia (AML-ETO9a), and myeloma (Vk*myc) (6, 7, 130, 143, 144). In addition, therapeutic effect of this vaccine approach was significantly enhanced when used in combination with immune checkpoint agonists, such as anti-4-1BB mAb (6). In other studies, the use of αGalCer-loaded tumor vaccines was also demonstrated to induce potent therapeutic responses against a murine model of MM (MOPC315.BM model) and found to generate long-term protection against tumor rechallenge (35). Interestingly, in a murine model of acute leukemia (C1498), the administration of αGalCer-loaded leukemic cells alone was found to be effective as a prophylactic vaccine but ineffective against established leukemia. The study found that while iNKT cells could be effectively activated, the downstream leukemia-specific T cell responses were suppressed. Instead, the benefit of vaccination became apparent following chemotherapy treatment, to prevent relapse of leukemia, and protect against rechallenge (21).

### Adoptive Transfer of iNKT Cells and CIK Cells

While the use of autologous cell-based vaccines has proven to be effective in animal models, a potential limitation in human patients is the high variability of iNKT cell frequency. Also, the functionality of iNKT cells often diminishes with tumor progression. Therefore, to circumvent this issue, adoptive transfer of activated and expanded iNKT cells derived from patient peripheral blood mononuclear cells (PBMCs) have been explored. Notably, CD3^+^CD56^+^ CIK cells, which represent a mixture of NK cell-like T cells, and incorporate an iNKT population, possess non-MHC-dependent tumor activity mediated through perforin and NKG2D expression (14, 15). By culturing autologous PBMCs under various conditions (e.g., αGalCer in the presence of GM-CSF and/or IL-2, or with a combination of cytokines such as IFNγ, OKT3, IL-2, and IL-15), *ex vivo* expansion of autologous activated iNKT/CIK cells from patients can be achieved (20, 30, 145). Successful expansion of functional iNKT cells from adult hematopoietic stem-progenitor cells using artificial APCs coated with CD1d-immunoglobulin (146, 147) as well as iNKT cell generation from induced pluripotent stem cells have also been explored (148). Adoptive transfer of *ex vivo* expanded iNKT cells in conjunction with αGalCer administration is an effective treatment against CD1d^+^ leukemic cells implanted in immunodeficient NOD/SCID mice (67). Similarly, adoptive transfer of iNKT cells activated *ex vivo* with IL-12 and IL-18 could initiate protection against lymphoma (ALC model) in mice (9). In humans, cultured iNKT/CIK cells are able recognize autologous or allogenic blood tumor cells *in vitro* (20, 30–32, 149). However, therapeutic use of *in vitro* expanded iNKT cells against blood cancers in humans is limited. Thus far, three phase I trials and a phase II trial have looked into the safety profile and efficacy of expanded activated autologous iNKT cells in patients with solid tumors (150–153). All of these studies demonstrated safety and feasibility of treatment as well as induction of IFNγ in circulating iNKT in patients. In the phase II study, αGalCer-loaded APCs administered alongside activated iNKT cells led to iNKT cell accumulation at tumor sites and some clinical efficacy in 50% of patients enrolled (153).

Notably, the use of expanded CIK cells in association with other treatments has led to complete cancer remissions in patients with hematological malignancies [reviewed in Refs. (14, 15)]. CIK cells have also been used in combination with HSCT in a bid to potentiate the overall inhibitory effects of GvHD in blood cancer patients receiving transplants (154). In a phase I study published by Luo et al. (154), patients enrolled were refractory to chemotherapy or had relapsed after early allogenic HSCT treatment. While some patients displayed a response to engraftment of donor cells, and infusion of CIK cells appeared to contribute to the prolonged survival in these patients, the overall efficacy of the combination treatment remains limited for this small cohort of patients with highly aggressive hematological malignancies (154). The extent to which these responses can be attributed to iNKT-like cells specifically, is unknown.

### Chimeric Antigen Receptor (CAR) Modified iNKT Cells and CIK Cells

Most recently, several studies have explored CAR engineering of iNKT/CIK cells (10, 22, 155). A summary of the proof of concept findings to date indicate that both CAR-NKT cells and CAR-CIK cells possess greater antitumor activity than their iNKT and CIK cell counterparts [recently reviewed in Ref. (156)]. In one example, donor CD62L^+^ iNKT cells that were identified to be highly proliferative *in vitro* were transduced with a CD19-specific CAR and tested for therapeutic activity against humanized mouse models of lymphoma and neuroblastoma. These CD62L^+^ CAR-NKT cells were demonstrated to persist long-term *in vivo* and were also highly effective at inhibiting tumor growth (10). The use of CAR-NKT cells was demonstrated to be safe and did not induce graft-versus-host disease (GvHD) in mice with neuroblastoma (155). In addition, the antitumor effects of CIK cells generated from donor PBMCs could also be further enhanced when transduced with CAR specific for CD19 and the CD28-CD3ζ signaling domain (22). These CAR-CIK cells were found to be highly effective against B-cell ALL (B-ALL) *in vitro*, including against CIK-resistant tumor cells. When tested *in vivo*, CAR-CIK cells were described to be more effective than non-CAR CIK cells in eliminating B-ALL tumors and promoting long-term survival in mice (22). We foresee that these studies will serve to accelerate research into modifying donor iNKT cells for adoptive therapies for blood cancers to complement other CAR-T cell-based therapies (157).

### Nanoparticle-Based Delivery Systems for iNKT Cell Adjuvants

To overcome some of the limitations associated with adoptive NKT cell-based approaches and to provide less costly and time-consuming alternatives for NKT cell-targeting immunotherapy, research into the use of nanoparticle-based systems are emerging [reviewed in Ref. (158)]. Briefly, nanoparticle vectors are delivery vehicles less than 1 µM in size and have wide applications in various diagnostic and treatment settings, including tumor immunotherapy (159). Delivery of glycolipid adjuvants in suitable nanoparticles presents several advantages over delivery in soluble form, such as reduced toxicity profile (owing to the reduced amount required to elicit a biological response), the ability to overcome iNKT cell anergy (160) and the preferential targeted delivery to APCs *in vivo* (158). To date, there exists various published studies in preclinical models of solid cancers on the nanoparticulate delivery of αGalCer alone or co-delivered with tumor-associated antigens (11, 161–164). By comparison, few therapeutic applications of nanoparticle delivery of glycolipid adjuvants have been reported for blood cancers. One such study utilized a targeted PLGA nanoparticle to codeliver a model tumor antigen ovalbumin (OVA) and αGalCer to DEC205^+^ CD8α^+^ DCs. iNKT cells were rapidly activated using this approach and could drive the induction of cytolytic tumor-specific CD8 T cells. When assessed in prophylactic and therapeutic settings against a model of thymoma, administration of targeted nanoparticles could significantly suppress early tumor growth (11). Recently, a liposomal form of αGalCer (RGI-2001) has been designed to circumvent GvHD after HSCT. Initial preclinical studies show that RGI-2001 could aid in graft-versus-leukemia effect and significantly prevented acute GvHD in lethally irradiated leukemia-bearing mice given allele-mismatched donor bone marrow cells or spleen cells. This effect was believed to be largely due to the enhanced expansion of donor-derived CD4^+^ regulatory T (Treg) cells that could exert its effects in an antigen-specific manner (165). Although RGI-2001 was demonstrated to induce expansion of NKT cells as well as higher IL-4 levels early after treatment, the correlation between NKT cell expansion and Treg induction was not clearly demonstrated. In a Phase II study in blood cancer patients, RGI-2001 was administered as a single dose in combination with HSCT. Similar to findings in mice, this study showed that RGI-2001 was generally tolerable in most patients and suggested that immunosuppressive Treg cells could be efficiently induced *in vivo* in a small proportion of patients. However, due to limited patient recruitment and difficulties in the detection of NKT cells in the blood in this particular study, the extent to which NKT cells contributed to overall GvL response remained inconclusive (89).

## Concluding Remarks

Increasing knowledge of how different blood cancers modulate their environment to avoid or suppress antitumor immunity has advanced the development of counteractive measures with immunotherapies. The fortuitous discovery of the potent NKT cell-stimulatory properties of αGalCer has enabled us to better understand how iNKT cells function to transactivate both the innate and adaptive immune system, and importantly, their unique role in antitumor immunity. However, encouraging findings in preclinical studies have not yet convincingly translated to similar outcomes in human cancers. In fact, the number of human trials testing the therapeutic use of various glycolipid compounds against cancer is limited, perhaps not only due to interindividual variability between patients but also due to the lack of understanding on the effects of tumors on decreasing iNKT frequencies and function. This is also true in harnessing the functions of NKT cells against GvHD after HSCT. In general, there still exists an uncertainty on the proper manipulation of iNKT cells and their different responses to a variety of glycolipids. We should continue to fully utilize preclinical models to understand how to best influence the functions of iNKT cells through synthetic glycolipid ligands, but also place more emphasis on the translation of these findings into the clinical setting, with the goal to rescue or enhance iNKT cell functions in different human blood cancer settings.

## Author Contributions

PL undertook critical review of the literature, wrote the manuscript, and designed the figure. MN contributed to the writing and editing of the manuscript. SM designed the scope of the manuscript and assisted with writing and editing of the manuscript.

## Conflict of Interest Statement

The authors declare that the research was conducted in the absence of any commercial or financial relationships that could be construed as a potential conflict of interest.
